# A Novel Communication Value Task Demonstrates Evidence of Response Bias in Cases with Presbyacusis

**DOI:** 10.1038/s41598-017-16673-y

**Published:** 2017-11-28

**Authors:** Mark A. Eckert, Kenneth I. Vaden, Susan Teubner-Rhodes, Brandon S. Bentzley

**Affiliations:** 1 Department of Otolaryngology – Head and Neck Surgery, Medical University of South Carolina, Charleston, USA; 2 Department of Psychiatry & Behavioral Sciences, Stanford University, Stanford, USA

## Abstract

Decision-making about the expected value of an experience or behavior can explain hearing health behaviors in older adults with hearing loss. Forty-four middle-aged to older adults (68.45 ± 7.73 years) performed a task in which they were asked to decide whether information from a surgeon or an administrative assistant would be important to their health in hypothetical communication scenarios across visual signal-to-noise ratios (SNR). Participants also could choose to view the briefly presented sentences multiple times. The number of these effortful attempts to read the stimuli served as a measure of demand for information to make a health importance decision. Participants with poorer high frequency hearing more frequently decided that information was important to their health compared to participants with better high frequency hearing. This appeared to reflect a response bias because participants with high frequency hearing loss demonstrated shorter response latencies when they rated the sentences as important to their health. However, elevated high frequency hearing thresholds did not predict demand for information to make a health importance decision. The results highlight the utility of a performance-based measure to characterize effort and expected value from performing tasks in older adults with hearing loss.

## Introduction

Older adults often report increased effort and fatigue when trying to understand speech, especially in challenging listening conditions. These difficulties persist even after accounting for audibility. Listening effort has been characterized as the tradeoff between the subjective value of understanding speech and the cost of implementing control^1,2^. Thus, examining the degree to which individuals value speech in different listening conditions can help explain hearing health behaviors. Here we describe the development of a web-based visual task for predicting hearing health behaviors that considers communication value based on how hard people will work to make a decision in a communication scenario.

Extracting meaning from degraded stimuli can be costly or effortful^3–7^. Questionnaires and dual-task measures have been used to characterize this effort. For example, the NASA Task Load Index questionnaire was used to show that older adults experienced significantly less perceived effort during consonant recognition when the stimuli were amplified by hearing aids compared to when the stimuli were not amplified^8^. These measures do not typically characterize the value in performing the task, however, which may affect the subjective experience of effort^9^. The Hearing Handicap Inventory for the Elderly (HHIE) indirectly measures value by asking questions about changes in potentially valuable experiences and activities, but it does not explicitly measure value from listening experiences.

Objective and quantitative measures of communication value are important because value-based decision-making can explain multiple findings in the hearing literature. The increased fatigue^10,11^, reduced cognitive function^12^, and increased illness^13^ related to elevated listening effort^2^ would increase the cost of listening to speech. The value that a listener experiences from a hearing-aid may diminish if there are minimal reductions in listening effort with hearing-aid use^10^. Thus, hearing-aid satisfaction can be influenced by the expected versus actual performance of hearing-aids^14–17^, which is consistent with the suggestion that patients who are skeptical of hearing-aid benefit are less likely to own hearing-aids^18^. Conversely, people who are willing to invest more time acclimating to their hearing-aids are more likely to experience satisfaction with their aid^19,20^.

Behavioral economic approaches provide explicit characterization of the expected value from a given behavior. For example, the classic Monetary Choice Questionnaire (MCQ)^21,22^ measures value using two-alternative forced choice questions. People choose between smaller immediate rewards versus larger delayed rewards (e.g., “Would you prefer $31 today, or $85 in 7 days?” Or, “Would you prefer $22 today, or $25 in 136 days?”). Holding the actual reward value constant, people are more likely to choose the immediate reward as the waiting time increases^22^. This delay or temporal discounting demonstrates that the propensity to choose a reward decreases as the cost of a behavior (e.g., waiting time) increases relative to its benefit (e.g., reward value). The MCQ has been effective in predicting behavior for health-related conditions^23–26^, in part because delay discounting characterizes the subjective value of a future reward. Thus, delay discounting may be sensitive to how much older adults with hearing loss will value future benefit from an aid^9^ and affect the decision to obtain a hearing-aid. Delay discounting may also be sensitive to how long older adults are willing to wait for hearing-aid benefit, which could affect hearing-aid satisfaction. In the context of experimental tasks, listeners who discount the value of a delayed reward would be expected to spend less time performing tasks^27,28^.

Like delay discounting, effort discounting involves a reduced propensity to choose a reward as cost increases, but cost is effort exertion rather than delay length^29^. The value of a reward can be measured by observing the consumption (demand) of a reward as a function of effort, i.e. cost^30^. When value is measured with this method, an individual who displays a high value for a reward is sometimes said to have a high demand for that reward^31^.

Here we defined communication value as the demand for information in a hypothetical health care listening scenario that we reasoned would be relatable for most people (please note that this definition of demand differs from task demand or task difficulty^32^). Prior work has included health-based rewards to assess effort discounting in younger and older adults^33^ and hypothetical scenarios (e.g., the MCQ) are commonly used for discounting studies because they can predict real-life discounting measures^34^.

We used a web-based presentation to examine how hard people would work to make decisions about sentences that were presented in text (i.e. communication value). This design was guided by paradigms that assess the degree to which the consumption of a reward (e.g., food or drug) changes with increasing cost (e.g., price or effort)^35^. This is similar to a listener asking someone to repeat speech that was difficult to understand. For example, an older adult in a narrative study of hearing-aid users described asking people to repeat what they said, but only when the “subject is important enough”^36^.

Participants were asked to decide if sentences from an administrative assistant or surgeon would be “Important” to their health (e.g., surgeon sentence: “Please do not take aspirin 24 hours before your surgery.”; administrative assistant sentence: “Can you confirm your date of birth for me?”). The sentences were presented in visual noise across different signal-to-noise ratios (SNR) to approximate the experience of listening in a noisy environment. We measured the frequency with which people viewed or re-glimpsed the briefly presented stimuli (875 ms) before making a health importance decision. They could also decide to quit a trial before making a health importance decision if they did not think it was possible to make a decision, particularly for sentences in low SNR conditions. This design allowed for the estimation of communication value for two speaker conditions with a different likelihood of health importance, where value was defined as the frequency of re-glimpsing the sentences with decreasing SNR.

The Communication Value Task was designed with visual stimuli rather than auditory stimuli so that it could be used to study listeners with a wide range of hearing loss without concerns about audibility of the stimuli affecting the measures. Like speech recognition, reading requires word identification and working memory abilities to understand the meaning of sentences as they unfold over time^37^. We also note that visual tasks have been used previously with success to study hearing^38,39^. In addition, discounting measures are generally considered to characterize relatively stable traits^40,41^, including temperament^42^, and general cognitive function^43^. The relation with cognition appears to be influenced, at least in part, by the function of pre-frontal cortex that integrates information from different modalities to guide behavior^44^. Thus, discounting measures of a domain general function obtained with visual stimuli would be expected to relate to audiologic measures.

Given the relatively limited extent of work on discounting in people with hearing loss, the focus of this project was to determine the extent to which to the Communication Value Task measures were related to individual variation in pure tone thresholds. We also determined the extent to which the Communication Value Task was related to a widely used measure of delay discounting to validate task measures as indices of discounting. Again, while discounting tasks appear to characterize relatively stable traits^40^, delay discounting can be relatively higher in older compared to middle-aged adults^33,45^. Changes in discounting could be due, at least in part, to age-related hearing loss^46,47^ and increased hearing handicap that reflects a diminished quality of life for older adults. We predicted that participants with greater delay discounting would demonstrate less demand for information to make a health importance decision because this behavior would increase the length of each trial and the task. The results described below show that hearing loss was associated with an increased expectation or bias to decide that information was important, but this behavior was not aligned with the demand for information to guide that decision.

## Materials and Methods

### Participants

Informed consent was obtainted from forty-seven middle-aged and older adults (mean age = 68.45, ± 7.84 years; 61% female) who participated in this Medical University of South Carolina (MUSC) Institutional Review Board approved study, which was performed in accordance with the Declaration of Helsinki. Participants were recruited and had their pure tone threshold data collected through ongoing hearing studies at MUSC. Three participants had unreliable delay discounting scores and were excluded^48^. Specifically, the difference in time and reward for each item of the MCQ (described below) explained less than 60% of the variance in their decisions^48^. In contrast, the same discounting task parameters explained an average of 95.43% (sd = 0.07) of the variance in item choices for the remaining 44 participants. Table 1 provides demographic information for the 44 participants whose results are reported in the current study.

**Table 1:** Sample characteristics and correlations between demographic, hearing and discounting variables.

	1. Age (years) 68.45 (7.84)	2. Sex (frequency) Female 61%	3. Low Frequency Thresholds 14.17 (8.83)	4. High Frequency Thresholds 35.33 (17.95)	5. HHIE (rank) 19.82 (18.98)	6. MCQ^ 0.49 (0.14)	7. Visual Perception (# correct) 12.88 (3.74)	8. Health Import (prop) 0.57 (0.180)	9. No Health Import (prop) 0.29 (0.17)	10. Quit Trials (rank prop) 0.14 (0.19)	11. Re-glimpse (rank #) 60.66 (47.95)
1.	1.00										
2.	0.10	1.00									
3.	0.03	0.28	1.00								
4.	0.53***	−0.39*	0.03	1.00							
5.	0.00	−0.06	0.26	0.40**	1.00						
6.	0.16	0.09	0.01	0.18	0.32*	1.00					
7.	−0.16	0.03	−0.17	−0.25	−0.20	−0.07	1.00				
8.	0.10	−0.26	−0.05	0.28	−0.02	−0.11	−0.02	1.00			
9.	−0.28	0.16	−0.22	−0.47***	−0.10	0.15	0.51***	−0.44**	1.00		
10.	0.16	0.11	0.25	0.16	0.11	−0.03	−0.44**	−0.58***	−0.47***	1.00	
11.	−0.02	−0.03	0.18	−0.11	0.13	0.34*	0.27	0.01	0.10	−0.10	1.00

**Figure 1:**
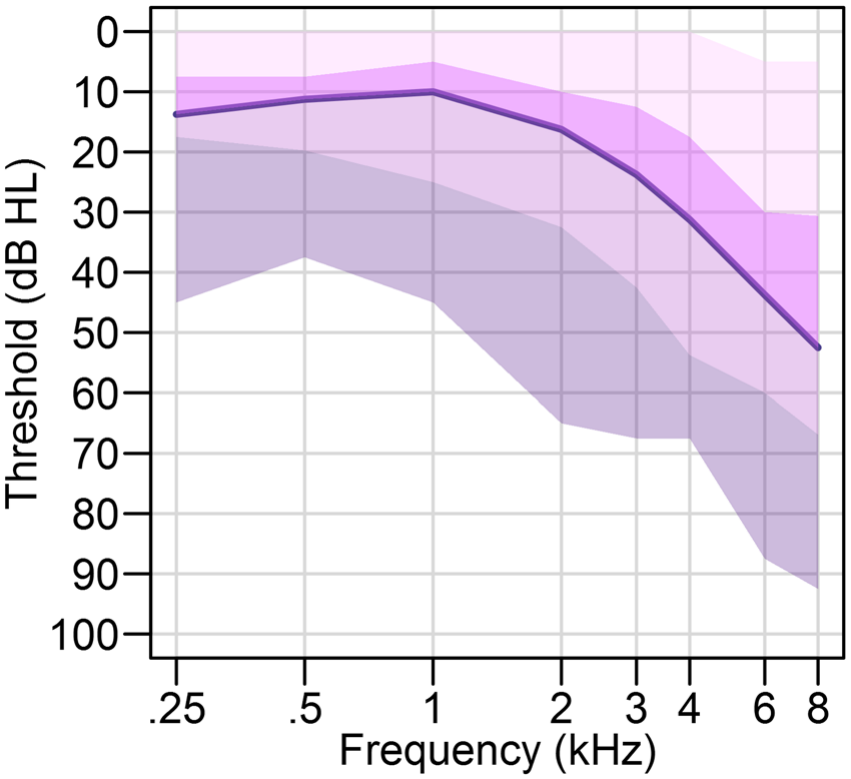
Quartile ranges for the pure tone thresholds across the sample. The dark purple line is the median for the sample.

***p < 0.001; **p < 0.01, *p < 0.05; Low and High Frequency Thresholds are the pure tone threshold constructs that are scaled with a mean of 0, but we present the average threshold for the low (250, 500, 1000 Hz) and high (2000, 3000, 4000, 6000, 8000 Hz) frequency thresholds. Also see Fig. 1 for the range of pure tone thresholds. MCQ, rank of the proportion of delayed reward choices from the Monetary Choice Questionnaire Delay Discounting; prop- proportion; Import- Importance. Frequency and means (std dev) are presented along the top row. ^A smaller proportion value indicates greater delay discounting.

### Pure Tone Threshold Measures

Figure 1 shows the quartile ranges and average pure tone threshold across standard audiometric frequencies (250–8000 Hz; Madsen OB922 audiometer and TDH-39 headphones) to demonstrate that thresholds ranged from normal to moderate hearing loss for the 44 participants. Participants provided their hearing thresholds and demographic information when performing our web-based tasks. Pure tone threshold data is typically collinear across lower and higher frequency thresholds. As in previous studies^49,50^, we created low and high frequency pure tone threshold variables that were averaged across ears. They were created using weights from a factor analysis of 1704 pure tone thresholds (250–8000 Hz; mean age = 69.92 years [SD = 7.24] that was 56% female; see Table 1 in^51^ for the factor analysis weights). To provide the reader a better understanding of the scaled values of these measures, a low frequency construct value of 0 corresponds to an approximate value of 17 dB HL for the average of 250 Hz and 500 Hz thresholds and a high frequency construct value of 0 corresponds to an approximate value of 41 dB HL for the average of thresholds from 1000 Hz to 8000 Hz.

The low and high frequency hearing constructs were unrelated (r_(42)_ = 0.03, ns) and accounted for 100% of the variance in the average pure tone threshold. Identification of two statistically unique pure tone threshold variables in older adults is consistent with the premise that there can be different mechanisms of age-related hearing loss that differentially affect low versus high frequency thresholds^51^. Thus, we were able to characterize the extent to which different patterns of hearing loss related to the Communication Value Task measures.

### Web-based Tasks

Participants performed: (1) the Communication Value Task; (2) a visual text recognition in noise task to control for individual differences in visual acuity that could affect performance on the Communication Value Task; (3) the MCQ; and (4) the HHIE^52^. jsPsych, a java script platform for delivering web-based experiments^53,54^, was used to collect data for this study. The code and stimuli for this experiment are available at www.eckertlab.org/tools.

#### Communication Value Task

Participants viewed sentences that were described as spoken by an administrative assistant or a surgeon during an outpatient clinic visit. The rationale for this design was to manipulate the potential health importance of information using a communication scenario that would be important to most people. The speaker information was presented in different SNR conditions so that we could examine 1) participant demand for information before making a health importance decision and 2) the extent to which this work was dependent on the expected health importance of the information (surgeon vs. administrative assistant). Demand was defined as the frequency of repeated viewing or re-glimpsing briefly presented sentences (875 ms) across SNR conditions.

One sentence was presented on each trial (n = 48) in one of two speaker conditions and six SNR conditions (24 trials for each of the 2 speakers; 8 trials for each of the 6 SNRs). The speaker conditions were presented in 4 blocks of 12 trials, with the 2 administrative assistant condition blocks that were book-ended by the 2 surgeon conditions. Each sentence appeared for 875 ms based on pilot testing to ensure that the sentences could be read and that participants would have to view the sentences multiple times, particularly in the low SNR conditions, to fully understand the text and make a health importance decision. SNR was manipulated by adding visual noise to the sentence text with the Matlab (MathWorks, Inc) imnoise function (speckle values of 0.15, 0.30, 0.45, 0.60, 0.75, 0.90).

**Figure 2:**
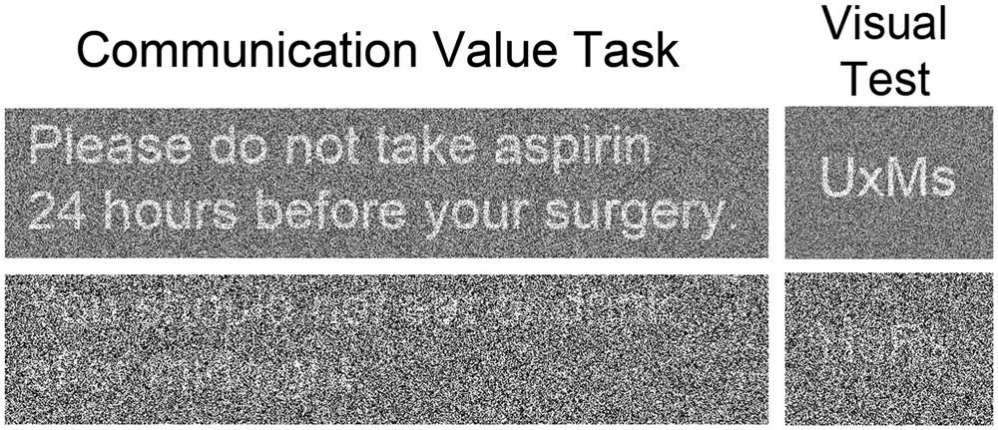
Stimulus examples. Communication Value Task sentences are presented for the surgeon condition with the highest and lowest SNR (e.g., “Please do not take aspirin 24 hours before your surgery”; “You should not eat or drink after midnight”). Participants responded using a key-press whether or not the information would be important to their health, to view the stimulus again, or to quit the trial and make no health importance decision. The same Matlab function was used to add noise to orthographic stimuli and vary SNR for a text recognition in noise test. Participants were instructed to type the upper- and lower-case letters that they could identify (e.g., UxMs; HdRk). The correctly typed letters were summed to produce a measure of text recognition in noise that required the same ability to the recognize words that were presented in the Communication Value Task.

The sentences are presented in Supplementary Table 1 and stimulus examples are presented in Fig. 2. There were no significant differences between administrative assistant and surgeon speaker condition sentences for word count (F_(1,47)_ = 0.76, ns), character count (F_(1,47)_ = 0.03, ns), or Flesch-Kincaid grade reading level (F_(1,47)_ = 1.46, ns). There were no significant sentence differences between SNR conditions for word count (F_(5,47)_ = 1.13, ns), character count (F_(5,47)_ = 1.47, ns), or Flesch-Kincaid grade reading level (F_(5,47)_ = 0.86, ns).

Key-press data were recorded during the task that indicated the response choice and latency for each trial. Key-presses represented whether: 1) the sentence included information “Important” to the participant’s health in this imaginary clinical scenario (Y key); 2) the information was “Not Important” to their health (N key); 3) the participant quit the trial (Q key); or 4) they decided to view again or re-glimpse the stimuli (R key). The importance ratings were used to confirm that the perceived importance of the information with the speaker and SNR conditions was successfully manipulated, while re-glimpsing provided an estimate of demand for information or value. We also examined decisions to quit a trial without making an importance decision as evidence of decision-making value.

Differences in response latencies between “Important” and “Not Important” decisions were examined to determine the extent to which the health importance ratings were affected by response bias, as in^55^. Specifically, response latency differences between two responses can indicate bias for the response with the shorter latency, because shorter latencies suggest that individuals needed less evidence before making their decision. Trials with outlier response latencies, defined as 2.5 SD from the mean of each participant’s response latency, were removed from this analysis. This affected an average of 3.16 ± 1.64 trials per participant.

#### Text Recognition in Noise Control Task

A visual text recognition in noise task was administered to control for differences in the ability to read the sentences in noise (Fig. 2). Across five trials, participants were instructed to use a keyboard to type the upper and lower case letters that were presented in the same Matlab-generated visual noise that was used to vary SNR for the Communication Value Task (stimulus/speckle value: UxMs/0.10; BeWi/0.30; tHvQ/0.50; PvCa/0.70; HdRk/0.90). Correctly typed letters were summed to obtain a text recognition in noise measure with a potential score of 20 (mean = 13.38 ± 2.38; range 8–18). Thus, there were no ceiling or floor effects. There was also a sufficient range in performance to examine individual differences in text recognition in noise.

#### MCQ

The MCQ is popular measure of delay discounting because of its ease of implementation, strong predictive power, and because real rewards produce similar discounting results^34^. Again, the MCQ includes 27 choices between smaller immediate rewards versus larger delayed rewards. The reward (money) and cost (time to reward) are varied to obtain a measure of discounting rate. We calculated the magnitude of delay discounting as the proportion of the delayed reward choices (i.e., money later versus now) relative to the total number of trials, as in^56,57^, because this quantity is intuitive to understand and nearly perfectly correlated with logistic regression and economic modeling estimates of discounting^57^. Lower discounting proportion values indicated greater delay discounting. This proportion measure was used to determine the extent to which delay discounting was related to the performance measures from the Communication Value Task.

#### HHIE

The 25-item HHIE was used to estimate the self-assessed social/situational (12 questions) and emotional (13 questions) consequences of hearing impairment (Ventry & Weinstein, 1982, Newman, *et al*., 1990). Standard scoring of the HHIE was performed [e.g., “Does a hearing problem cause you to use the phone less often than you would like?” Yes (4); Sometimes (2); No (0)]. Responses to the items were summed to produce a total HHIE score (mean = 19.81, ± 18.98; range = 0–74).

### Statistical Analyses

Logistic regression analyses were performed using generalized linear mixed models (GLMM; R version 3.2.3 R-package lme4 version 1.1.12) to examine how the experimental conditions, pure tone thresholds, and delay discounting measures influenced decision-making (*D*) on each trial (*t*). Three separate GLMMs were performed to examine influences on 1) “Important” vs “Not Important” decisions, 2) quitting a trial vs all other response options (i.e., quit vs “Important”, “Not Important”, or re-glimpse), and 3) sentence re-glimpsing vs all other response options. *SNR* and *speaker conditions*, as well as their interaction, the *pure tone threshold constructs*, and MCQ *delay discounting* were included as independent variables. In summary, the logistic regressions can be described as *Dt* = *SNR* * *Speaker* + *Low Frequency Threshold Construct* + *High Frequency Threshold Construct* + *Delay Discounting* + (1|*Participant*) + *error*. The effects for each independent variable are described below using Z-scores. Regression and t-test comparisons were used to clarify the relative independence of significant associations and clarify GLMM results. Data were rank-ordered when there was evidence of a non-normal distribution (e.g., the HHIE scores).

## Results

Descriptive statistics and correlations between the demographic, hearing, and discounting measures in Table 1 show that the sample was composed of typical middle-aged and older adult participants. The oldest adults had the most elevated high frequency hearing thresholds. In addition, participants selected the more immediate reward for approximately half of the MCQ trials, which is within the same range as reported in a study of 111 young to middle-aged adults^57^.

### Communication Value Task Design Effects

#### SNR Effects

SNR was a significant predictor for each GLMM designed to examine how experimental conditions influenced health importance ratings, re-glimpsing, and quit decisions. Specifically, participants more frequently rated sentences as “Important” to their health, re-glimpsed a sentence before making a health importance decision, and quit a trial when a sentence was presented in a lower SNR (i.e., noisier) condition (Table 2). SNR was the only task manipulation that affected the decision to quit a trial.

**Table 2:** Results from 3 GLMM Analyses to show experimental design effects and individual differences that predicted Communication Value Task performance (Z scores).

Predictors	Important to My Health or Not		Quit a Trial		Re-glimpsed	
(Intercept)	1.64		−1.44		3.57	***
Speaker Condition	−1.97	*	−0.09		2.32	*
SNR	−7.40	***	−11.65	***	−9.21	***
SNR * Speaker Condition	7.99	***	−0.92		−2.31	*
Low Frequency Pure Tone Threshold Construct	1.30		1.32		1.89	~
High Frequency Pure Tone Threshold Construct	3.40	***	0.39		−1.40	
MCQ Delay Discounting	−1.92	~	−0.68		2.66	**

^~^p < 0.10; *p < 0.05; **p < 0.01; ***p < 0.001; The influence of visual text recognition was examined for signficant effects, as described in the text, but was not included in the GLMM because of convergence errors.

#### Speaker and SNR Interaction

**Figure 3:**
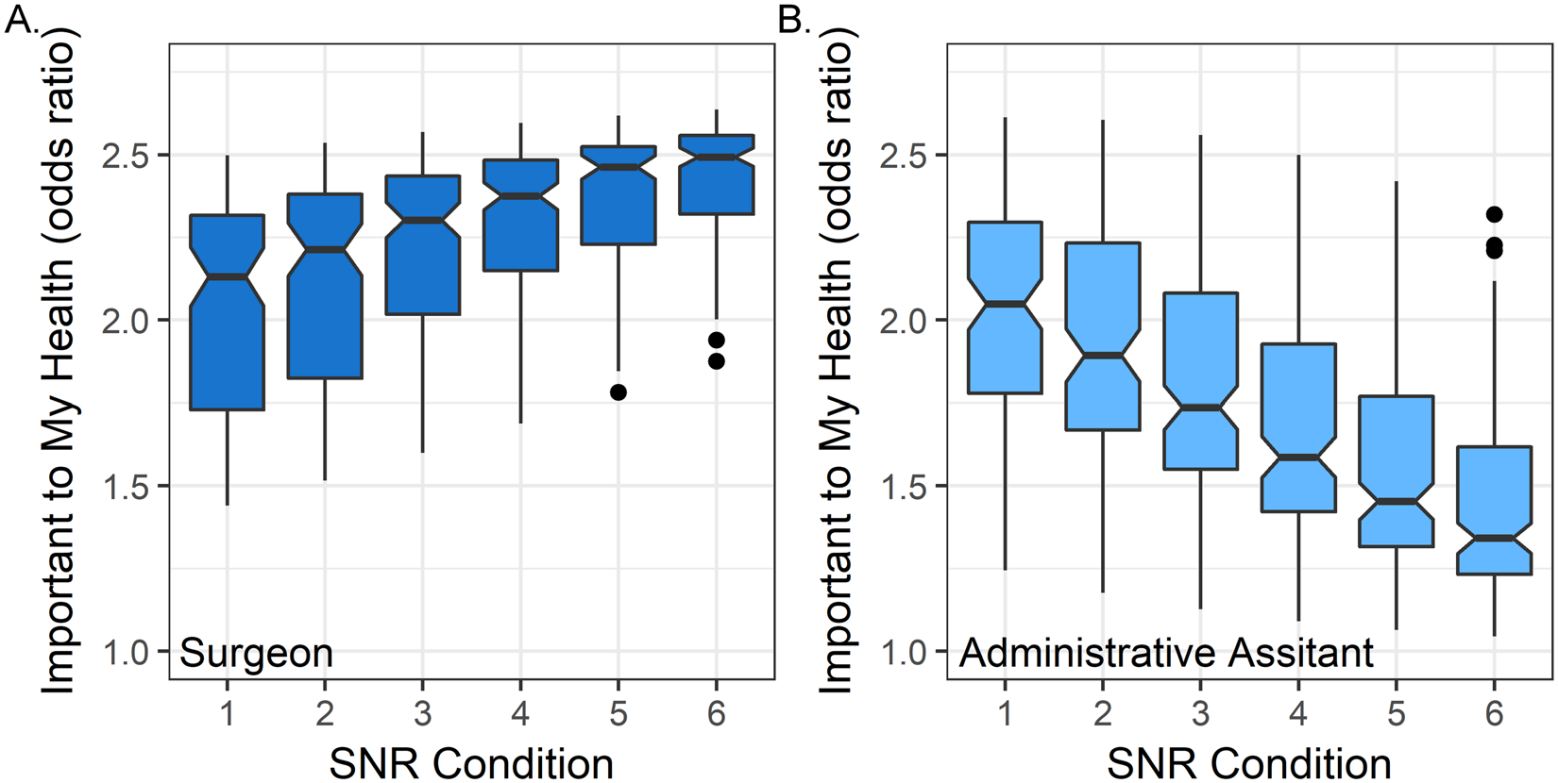
Speaker and SNR conditions interact to influence decision-making. (**A**) The odds ratio of participants rating the sentences as being “Important to My Health” compared to quit or “Not Important” increased with SNR for the surgeon condition. (**B**) The odds ratio of “Important to My Health” ratings compared to quit or “Not Important” increased with decreasing SNR for the administrative assistant condition. Odds ratios were obtained using an exponential conversion of the fitted effects coefficients from the GLMM.

The GLMM analyses also revealed that significant effects of speaker condition were observed for the health importance ratings and decision to re-glimpse the sentences. The significant difference in frequency of “Important” ratings between speakers was largely due to the highest SNR conditions. This result was validated by a significant interaction between speaker condition and SNR (Table 2), which emerged because ratings of health importance were similar for the surgeon and administrative assistant conditions when text intelligibility was low, but were higher for the surgeon than the administrative assistant when the text was clear (Fig. 3A,B). These results suggest that participants were more likely to rate sentences as “Important” when they were uncertain about the meaning of the sentences in the low SNR conditions.

While the GLMM results indicated that re-glimpsing was most strongly influenced by SNR (Table 2), additional inspection of the results indicated that there was an interaction between speaker, SNR, and importance decisions. For the two most intelligible SNR conditions, paired sample t-tests of the number of re-glimpses revealed that participants more frequently re-glimpsed the surgeon compared to the administrative assistant sentences before making an “Important” decision (SNR 6 “Important” sentences: t_(43)_ = 2.78, p = 0.008; SNR 5 “Important” sentences: t_(43)_ = 1.79, p = 0.080) and more frequently re-glimpsed the administrative assistant compared to the surgeon sentences before making a “Not Important” decision (SNR 6 “Not Important” sentences: t_(43)_ = −5.23, p = 0.000005: SNR 5 “Not Important” sentences: t_(43)_ = −4.26, p = 0.0001). There were no significant re-glimpse differences between speaker conditions in the lowest SNRs. Thus, participants appeared to use re-glimpsing in the most intelligible conditions to confirm an expectation about the health importance of the speaker sentences.

### Hearing Loss Relates to Increased Response Bias

The GLMM analyses demonstrated that participants with elevated high frequency hearing thresholds more frequently button-pressed to indicate that information from an administrative assistant and surgeon would be “Important” to their health rather than “Not Important” (Table 2), which as we describe below was largely due to fewer “Not Important” decisions with increased high frequency hearing loss. This relation between hearing loss and importance rating remained significant when age was included the high frequency hearing variable in the GLMM.

**Figure 4:**
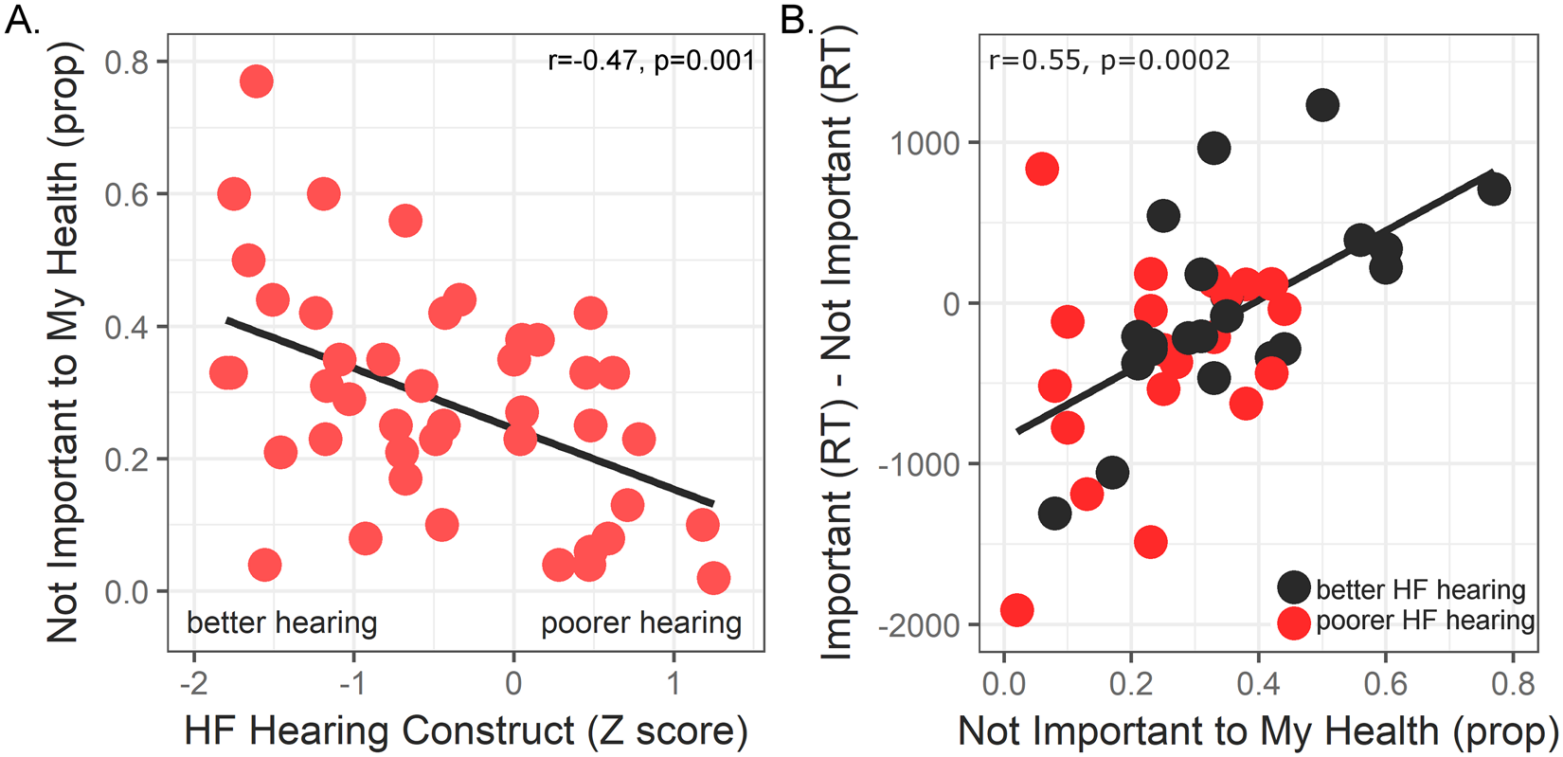
High frequency (HF) hearing loss predicts decision-making and response bias. (**A**) Participants less frequently rated the sentences as being “Not Important to My Health” when they had elevated HF thresholds. The HF hearing Z-score value of 0 corresponds to an approximate average value of 41 dB HL for pure tone thresholds from 1000 to 8000 Hz. (**B**) Participants with a lower proportion of “Not Important to My Health” ratings, again exhibited more HF hearing loss (red circles: HF Z-score median split), and were more likely to take more time to decide that information was “Not Important to My Health” than their more frequent “Important to My Health” decision. Negative values reflect faster reaction times for “Important to My Health” than “Not Important to My Health” decisions, and thus indicate a bias to respond that the information is “Important to My Health”. prop = proportion; RT = reaction time or response latency.

“Important” and “Not Important” ratings across trials were not inverse measurements because of the option to quit a trial. There appeared to be greater sensitivity to individual differences for the “Not Important” decision when aggregating data across trials. For example, Table 1 shows that high frequency hearing threshold, age, and text recognition in noise were significantly related to “Not Important” ratings. Multiple regression demonstrated that elevated high frequency hearing thresholds significantly predicted fewer “Not Important” decisions, after accounting for the effects of age and text recognition in noise (Multiple R = 0.64, p = 0.0001; high frequency threshold variable Std Beta = −0.36, p = 0.020; low frequency threshold variable Std Beta = −0.14, ns; age Std Beta = −0.02, ns; text recognition in noise Std Beta = 0.39, p = 0.004). The relation between “Not Important” and high frequency hearing is shown in Fig. 4A.

The hearing loss associations with health importance ratings suggested the possibility that response bias could explain these results. To examine the possibility that older adults with elevated high frequency hearing thresholds were more likely to exhibit response bias^55^, we examined the average response latency for “Important” and “Not Important” decisions. As shown in Fig. 4B, participants with elevated high frequency hearing thresholds took longer to decide that information was “Not Important” than when deciding information was “Important” [high frequency hearing by response latency (“Important” – “Not Important”): r_(39)_ = −0.35, p = 0.023]. Hierarchical multiple regression showed that the high frequency hearing construct was no longer predictive of the response latency estimate of response bias when the proportion of “Not Important” ratings was included in the model (Table 3). Older adults with fewer “Not Important” ratings exhibited a longer reaction time for their “Not Important” ratings compared to their “Important” ratings. In other words, the higher frequency of “Important” ratings in participants with hearing loss appeared to reflect decision-making bias based on the shorter response latencies for these decisions.

**Table 3:** Hierarchical multiple regression demonstrates that the difference in response latencies for important and not important decisions was significantly associated with high frequency hearing and frequency of “Not Important” decisions.

Level	Multiple R	Variable	Std Beta	t score	p value
1	0.354	Constant		−2.88	0.006
		High Frequency Pure Tone Threshold	−0.35	2.36	0.023
2	0.369	Constant		0.16	ns
		High Frequency Pure Tone Threshold	−0.38	2.44	0.019
		Visual Perception: Text Recognition	−0.11	0.71	ns
3	0.638	Constant		0.57	ns
		High Frequency Pure Tone Threshold	−0.09	−0.62	ns
		Visual Perception: Text Recognition	−0.35	−2.44	0.02
		“Not Important to My Health” Decision	0.66	4.11	0.0002

### Discounting Predicts Performance Effort and Not Hearing Loss

**Figure 5:**
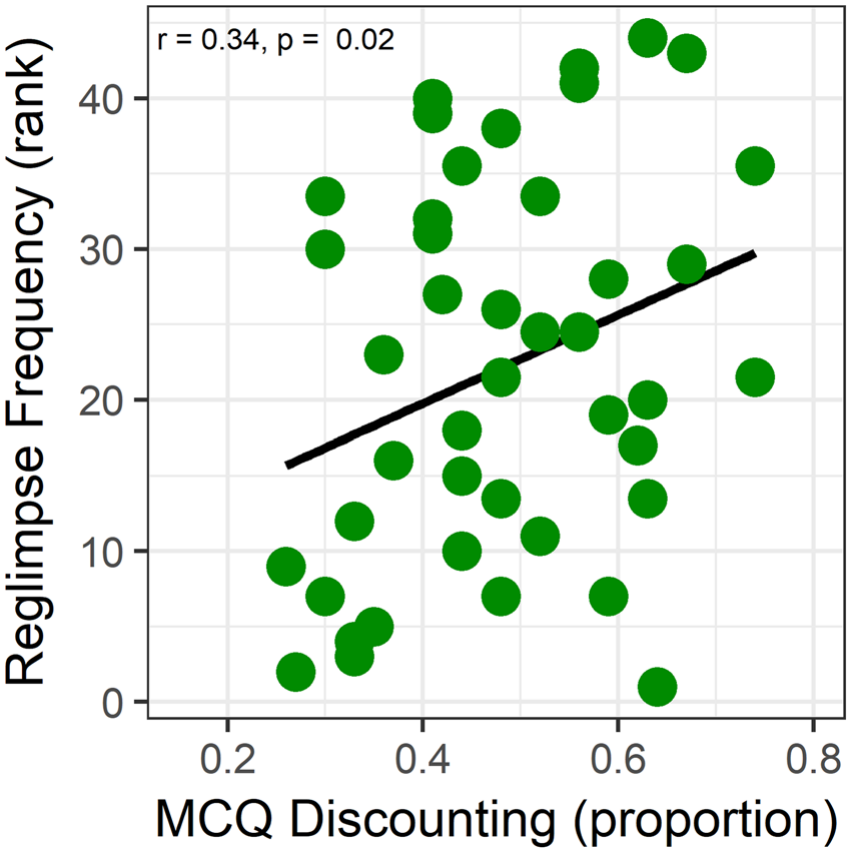
The proportion of delayed reward choices from the MCQ delay discounting measure increased with the frequency of re-glimpsing or viewing a sentence again before making a health importance decision or quitting the trial during the Communication Value Task (95% CI −0.58 to −0.05). Frequency of re-glimpsing was rank-ordered from lowest to highest to correct for a non-normal distribution.

Participants with higher delay discounting, or cases who would prefer a smaller immediate reward compared to a larger delayed reward, re-glimpsed each sentence fewer times than participants with lower delay discounting (Tables 1 and 2; Fig. 5). This effect was present for both speaker conditions (surgeon: r_(42)_ = −0.35, p = 0.019; administrative assistant: r_(42)_ = −0.30, p = 0.046). Table 4 presents the results of a hierarchical multiple regression, which demonstrates that this discounting effect was significant even after controlling for text recognition in noise and the high frequency hearing threshold construct. These results are consistent with the premise that participants who are less willing to wait for larger reward would exhibit lower demand for information.

**Table 4:** Hierarchical multiple regression demonstrated that delay discounting (MCQ) was uniquely associated with re-glimpsing or the frequency of viewing the Communication Value Task sentences.

Level	Multiple R	Variable	Std Beta	t score	p value
1	0.344	Constant		4.60	0.000
		MCQ	0.34	2.37	0.022
2	0.425	Constant		4.77	0.000
		MCQ	0.37	2.57	0.014
		Low Frequency Pure Tone Threshold Construct	0.18	1.25	ns
		High Frequency Pure Threshold Tone Construct	−0.18	−1.24	ns
3	0.515	Constant		1.03	0.311
		MCQ	0.38	2.73	0.009
		Low Frequency Pure Tone Threshold Construct	0.23	1.65	ns
		High Frequency Pure Threshold Tone Construct	−0.11	−0.75	ns
		Visual Perception	0.31	2.13	0.040

Finally, delay discounting was not significantly associated with the pure tone threshold measures (Table 1), but did exhibit a modest relationship with hearing handicap (Table 1; Spearman’s rho 95% confidence interval = 0.06 to 0.58). Participants reporting more hearing handicap exhibited less delay discounting. While this relationship was significant after accounting for low and high frequency pure tone thresholds (partial r = −0.31, p = 045), we cautiously interpret this result as evidence that cases with more hearing handicap will wait for a larger and delayed reward compared to choosing an immediate reward.

## Discussion

The Communication Value Task was designed to measure the demand for information to make a decision about health care listening scenarios across different speaker and SNR conditions. Our goal was to measure a trait that influences performance across different types of tasks and that could be used to provide an objective measure of effort in people with hearing loss. In support of this goal, delay discounting significantly predicted how hard participants worked to read and make a health importance decision about sentences in noise. Participants with elevated pure tone thresholds in the high frequencies more frequently reported that information would have health importance. However, people with hearing loss did not exhibit evidence of working harder to make a health importance decision. This dissociation highlights the significance of obtaining behavioral measures of expected value because self-report may not always be indicative of value when there is potential for subjective bias. These results suggest that the Communication Value Task measures different dimensions of decision-making that have relevance to understanding hearing health behaviors.

### Rationale for Experimental Design

Evidence that expected value can affect hearing-aid use and outcomes^13–16^ and the absence of performance-based discounting tasks for hearing studies provided the motivation for developing a measure to predict hearing health behaviors, such as the frequency of hearing aid use. In developing this task, we considered evidence that speech recognition in noise engages neural systems that estimate the expected value from optimizing task performance with increasing task difficulty^58^ or lower SNR^1^. For these reasons, we wanted a measure of expected value from communication and chose to define value as the demand for information to make a health importance decision across SNR conditions. Specifically, the demand or re-glimpsing measure is conceptually similar to asking speakers to repeat what they said in difficult listening conditions. The likelihood that someone interrupts a speaker to repeat can depend on the expected value of the information conveyed, particularly among older adults with hearing loss^36^.

Manipulating the visual SNR did require participants to work harder in the low SNR conditions to make a decision. Moreover, re-glimpsing was higher in cases with lower MCQ delay discounting. In other words, the cost/benefit decision-making that is characterized by the MCQ predicted how hard people would work to make an informed decision. To the extent that our measure of demand reflects effort discounting, this association with the MCQ appears to be consistent with modest associations between delay and effort discounting^33,59^.

The results of the current study may explain why delay and effort discounting measures can exhibit modest associations. Re-glimpsing increased the length of time that participants spent on each trial, which lengthened duration of the task. Thus, effort discounting tasks where effort increases the duration of the task would be expected to exhibit stronger associations with measures of delay discounting.

Importantly, age, sex, or hearing loss were not significantly associated with re-glimpsing or delay discounting. These results indicate that the demand for information that is measured with the Communication Value Task can vary across older adults, including older adults with elevated pure tone thresholds. There was a modest relation between self-reported hearing-handicap and delay discounting, which was significant after accounting for hearing thresholds. This observation may be consistent with evidence that older adults with high hearing-handicap also report increased speech recognition effort and frustration^60^, perhaps because they are investing more time to recognize speech. Future studies will examine how variation in these behavioral economic measures predict hearing aid ownership, frequency/duration of use, and satisfaction.

### Hearing Loss and Increased Response Bias

The Communication Value Task was sensitive to response bias, particularly for the administrative assistant sentences with decreasing SNR. There is evidence that response bias increases with increasing stimulus uncertainty in perceptual decision-making tasks, where the direction of the bias is determined by cues and/or priors that provide information about the likelihood of an event^61^. For example, response caution was observed when participants were asked to detect an auditory stimulus that occured without a cue compared to when the timing of presentation was cued^62^. In the current study, response bias depended on the speaker condition and SNR condition. For example, the decision that surgeon sentences would be not important to one’s health appeared to involve more caution because of the understanding that the hypothetical listening scenario involved a health care setting.

Response biases during perceptual decision-making tasks have been observed to change with age in studies of older adults. For example, older adults exhibited a positive bias in reporting that they had recognized words in noise compared to younger adults during a word recognition in noise task^63^. Older adults also were more likely to report hearing a word rather than a non-word compared to younger adults^64^. The authors suggested that bias for word judgements provided a performance strategy benefit because it occurred with a tendency to recognize words more quickly. A similar strategy could explain why the bias results are in the opposite direction of a larger literature suggesting that older adults exhibit increased response caution based on response latency data^65^.

In many experiments, older adults slow their behavior to optimize performance^66^. The speed – accuracy tradeoff for a given task may determine the response bias that older adults demonstrate. In the current study, there was response latency evidence that older adults with elevated high frequency hearing thresholds took less time to make “Important” decisions and took more time to make “Not Important” decisions. Response latency differences between conditions can result from response preferences^67^, which make non-preferred responses relatively more effortful and slower^55^. Together with the findings described above, this pattern of response bias guides the interpretation that participants with high frequency hearing loss were biased to make a judgement that met an expectation based on the health care context of the experiment.

But why does response bias increase with age-related high frequency hearing loss? This response bias effect did not appear to be due to a co-morbid visual impairment as the measure of text recognition in noise did not predict response latency differences between health importance choices. Older adults with hearing loss may rate the sentences as having health importance because of other comorbid health conditions and related health care visits and expenses^68,69^ that increase the perceived importance of information in a health care setting. In this context, the bias towards deciding that health information is important could reflect a management or balancing of choices to minimize error and improve decision-making^70^. Here, older adults with high frequency hearing loss and co-morbid health problems may have better health care outcomes if they are biased to consider more information in a clinical setting as important to their health. While a limitation of this study is the absence of data about co-morbid health conditions, the hearing loss and bias results were independent of age differences and age would be expected to covary with comorbid health conditions.

One additional explanation for the hearing loss and bias finding, which is based on the response bias literature described above, is that the daily experience of hearing difficulty increases a dependence on a response bias strategy across different types of perceptual tasks. This bias could stem from the expectation that working harder does not always provide benefit when information is too difficult to perceive and thus serves as strategy to conserve energy. While somewhat speculative, this interpretation is supported by evidence that the high frequency hearing threshold and importance rating was strongest in the less intelligible SNR conditions. Future study of co-morbid health conditions and health care experiences is necessary to resolve the question as to why high frequency hearing loss was associated with increased response bias.

Finally, we also interpreted the hearing loss and elevated frequency of “Important” decisions or decreased frequency of “Not Important” decisions to reflect response bias because this association occurred in the absence of increased re-glimpsing to make an informed decision, particularly when the stimuli were difficult to perceive. These results indicate that the expected importance of information may not always align with how hard people are willing to work when stimuli are difficult to perceive.

### Conclusions and Future Directions

We developed a performance-based measure of communication value that assesses the demand for information or how hard people are willing to work to make health importance decisions. There were two primary results of interest. First, the task appears to characterize a behavioral economic discounting construct based on the frequency with which people re-glimpse the briefly presented stimuli. Second, the task appears to characterize response bias that was most pronounced in people with high frequency hearing loss. While these results were independent of variation in text recognition in noise for this visual task, studies involving auditory stimuli are necessary to determine the specificity of the results and further establish the applicability of the Communication Value Task to understanding hearing health behaviors.

## Electronic supplementary material

Supplemental Table 1
