# Supplementary material for: A Novel Communication Value Task Demonstrates Evidence of Response Bias in Cases with Presbyacusis
Source: Sci Rep. 2017 Nov 28;7:16512. doi: 10.1038/s41598-017-16673-y (PMC5705661; doi:10.1038/s41598-017-16673-y)
Supplement: Supplementary file 1 — Supplemental Table 1 [file 41598_2017_16673_MOESM1_ESM.docx]

Supplementary Materials for **A Novel Communication Value Task Demonstrates Evidence of Response Bias in Cases with Presbyacusis**

Mark A. Eckert^1^, Kenneth I. Vaden Jr.^1^, Susan Teubner-Rhodes^1^, and Brandon S. Bentzely^2^

^1^Department of Otolaryngology – Head and Neck Surgery, Medical University of South Carolina

^2^Department of Psychiatry & Behavioral Sciences, Stanford University

Address correspondence to Mark A. Eckert, Hearing Research Program, Department of Otolaryngology – Head and Neck Surgery, Medical University of South Carolina. MSC 550, Charleston, S.C. 29425-5500, eckert@musc.edu

**Supplemental Table 1. The 48 sentences that were presented during the Communication Value Task.**

| **Condition** | **Sentences** |
| --- | --- |
| Surgeon | You should expect to feel sore. |
|  | You should get out of bed and walk as soon as possible. |
|  | Please do not take aspirin 24 hours before your surgery. |
|  | You can resume regular activity after you leave the hospital. |
|  | Do you feel weak? |
|  | Please call us if you develop a fever. |
|  | Have you had a surgery before? |
|  | Call us if you have any questions before surgery. |
|  | We will check your body temperature before surgery. |
|  | It will take about six hours to complete your surgery. |
|  | You may experience a loss of sensation. |
|  | You will wake up in a private room after the surgery. |
|  | Please let the nurse know if you want to get out of bed |
|  | The nurse will call your spouse with updates during surgery |
|  | It is important to take your medications. |
|  | How are you feeling today? |
|  | Keep your incision site clean and dry. |
|  | We will give you prescriptions for your medications. |
|  | We will reschedule your surgery if you have a high temperature. |
|  | Are you allergic to any medications? |
|  | You can expect to be in the hospital for two days after surgery |
|  | You need to arrive for your surgery early in the morning. |
|  | You are likely to have a scar from the procedure. |
|  | You should not eat or drink after midnight. |
| Administrative Assistant | Your private health information is protected. |
|  | Please write your name and appointment time on this sheet |
|  | Please wait a moment while I help this other patient. |
|  | Please follow me to the waiting room. |
|  | We will call your name when the doctor is ready to see you. |
|  | The doctor will be in to see you soon. |
|  | What is your home address? |
|  | This form explains laws about your health information. |
|  | The doctor would like you to return the day before your surgery. |
|  | Please list an emergency contact person. |
|  | We need to schedule your next visit. |
|  | Please complete the forms in the patient waiting area. |
|  | Why do you have an appointment today? |
|  | Answer the questions on this form about your medical history. |
|  | I need to review your information with you. |
|  | Please list your allergies on this form |
|  | Can you confirm your date of birth for me? |
|  | Is this a general check up? |
|  | Are you a first time patient? |
|  | I need to see your ID to confirm your information. |
|  | May I please see your insurance card? |
|  | You need to pay the insurance co-payment. |
|  | We need your approval to share your findings with other doctors. |
|  | There is also a privacy agreement form for you to read and sign. |
